# When the Tube Tells the Tale: A Visual Cue Reveals a Rare Cause of Acute Pancreatitis

**DOI:** 10.7759/cureus.101838

**Published:** 2026-01-19

**Authors:** Claud Bugheni, Vijay Reddy

**Affiliations:** 1 Emergency Medicine, Augusta University Medical College of Georgia, Augusta, USA

**Keywords:** diagnostic medicine, emergency medicine physician, hypertriglyceridemia (tg), hypertriglyceridemic pancreatitis, visual methods

## Abstract

Acute pancreatitis is a frequent cause of abdominal pain in the emergency department, but identifying less common triggers - such as hypertriglyceridemia - requires careful clinical observation. In this case, a visual clue during phlebotomy - a milky, lipemic appearance of the patient’s serum - prompted targeted testing that revealed severely elevated triglycerides. Since lipid panels are not routinely ordered in ED patients with abdominal pain, such subtle findings can be critical for early diagnosis. Prompt recognition is key, as the management of hypertriglyceridemia-induced pancreatitis differs significantly from more common etiologies and often includes insulin infusion or other triglyceride-lowering therapies.

## Introduction

Acute pancreatitis is a common gastrointestinal emergency with etiologies that vary widely, including gallstones, alcohol use, medications, and metabolic disturbances [1-3]. Among these, hypertriglyceridemia is an infrequent but well-documented cause, accounting for approximately 1-4% of cases in the general population [4-6]. When triglyceride levels exceed 1,000 mg/dL -particularly above 2,000 mg/dL - the risk for pancreatitis rises sharply, thought to be mediated by excess chylomicron accumulation, lipotoxicity, and ensuing pancreatic inflammation [3].

Although hypertriglyceridemia-induced pancreatitis (HTGP) is often associated with poorly controlled diabetes or familial dyslipidemias, it can present in a wide clinical spectrum, ranging from mild abdominal pain to fulminant systemic illness. Prompt recognition is essential, as early initiation of triglyceride-lowering therapy - typically insulin infusion, with or without plasmapheresis - has been shown to reduce complications and shorten hospital stays [5-7].

We present a case of a 41-year-old woman with HIV and comorbid hyperlipidemia who was admitted with acute pancreatitis attributed to severe hypertriglyceridemia. This case underscores the importance of clinical vigilance and the diagnostic value of keen physical and visual observations. In this instance, the striking appearance of lipemic blood in a collection tube led to an early diagnosis and timely intervention.

## Case presentation

A 41-year-old black female with a medical history significant for HIV (currently on Biktarvy), hypertension, hyperlipidemia, gastroesophageal reflux disease, and palpitations presented to the emergency department with acute epigastric abdominal pain and was admitted to the Medical Intensive Care Unit (MICU) for suspected acute pancreatitis.

**Figure 1:**
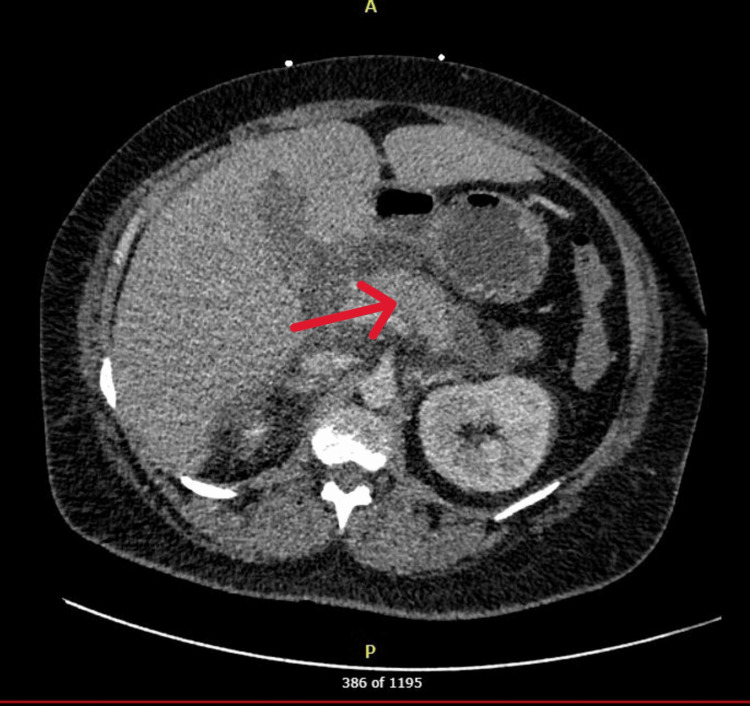
CT scan of acute pancreatitis from case report Radiology report of CT scan. Edematous appearance of the pancreas with severe surrounding inflammatory changes with fluid tracking in the mid abdomen, right paracolic gutter, and right greater than left anterior pararenal spaces. Mild inflammatory changes in the right perirenal space. Findings consistent with acute interstitial edematous pancreatitis indicated by the red arrow.

On initial examination, the patient was afebrile but tachycardic, hemodynamically stable, and maintained adequate oxygen saturation on room air (Table 1). Laboratory evaluation revealed leukocytosis, anemia, hyponatremia, metabolic acidosis, hypokalemia, and severely elevated triglycerides. Lipase was moderately elevated, while liver enzymes remained within normal limits. CT imaging of the abdomen and pelvis was consistent with acute pancreatitis (Figure 1) [8].

**Table 1:** Patient encounter vitals and lab results

Variables	Normal Range	Patient
HR	60-100 bpm	~110 bpm
WBC	3.4-10.8 x 10E3/uL	11.1 K/µL
Hemoglobin	11.1-15.9 g/dL	10.6 g/dL
Sodium (Na)	135-145 mmol/L	122 mmol/L
Potassium (K)	3.5-5.5 mmol/L	2.3 mmol/L
Triglyceride	0-149 mg/dL	4573 mg/dL
Lipase	6-51 U/L	290 U/L
Bicarbonate	20-31 mmol/L	10 mmol/L

**Figure 2:**
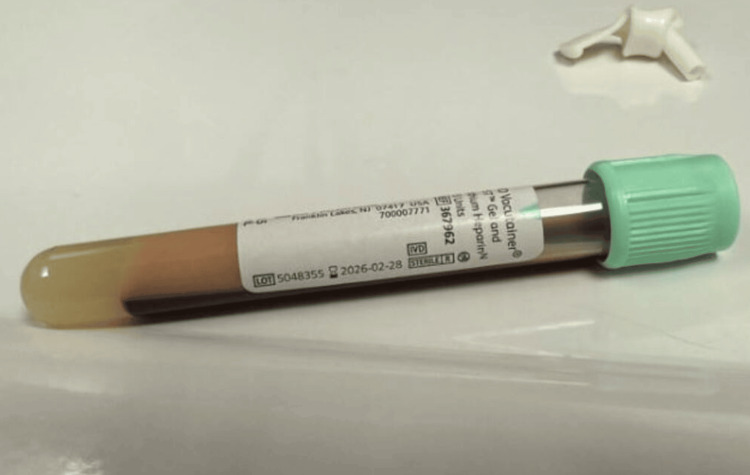
Lipemic appearing blood draw

Notably, a striking visual observation was made during the blood draw: a dense, creamy supernatant separated from the patient's serum in the collection tube (Figure 2) - prompting suspicion for significant hyperlipidemia. Although triglyceride levels are not routinely ordered in the ED for abdominal pain, this finding led to a targeted lipid panel that confirmed severe hypertriglyceridemia (TG 4573 mg/dL).

**Figure 3:**
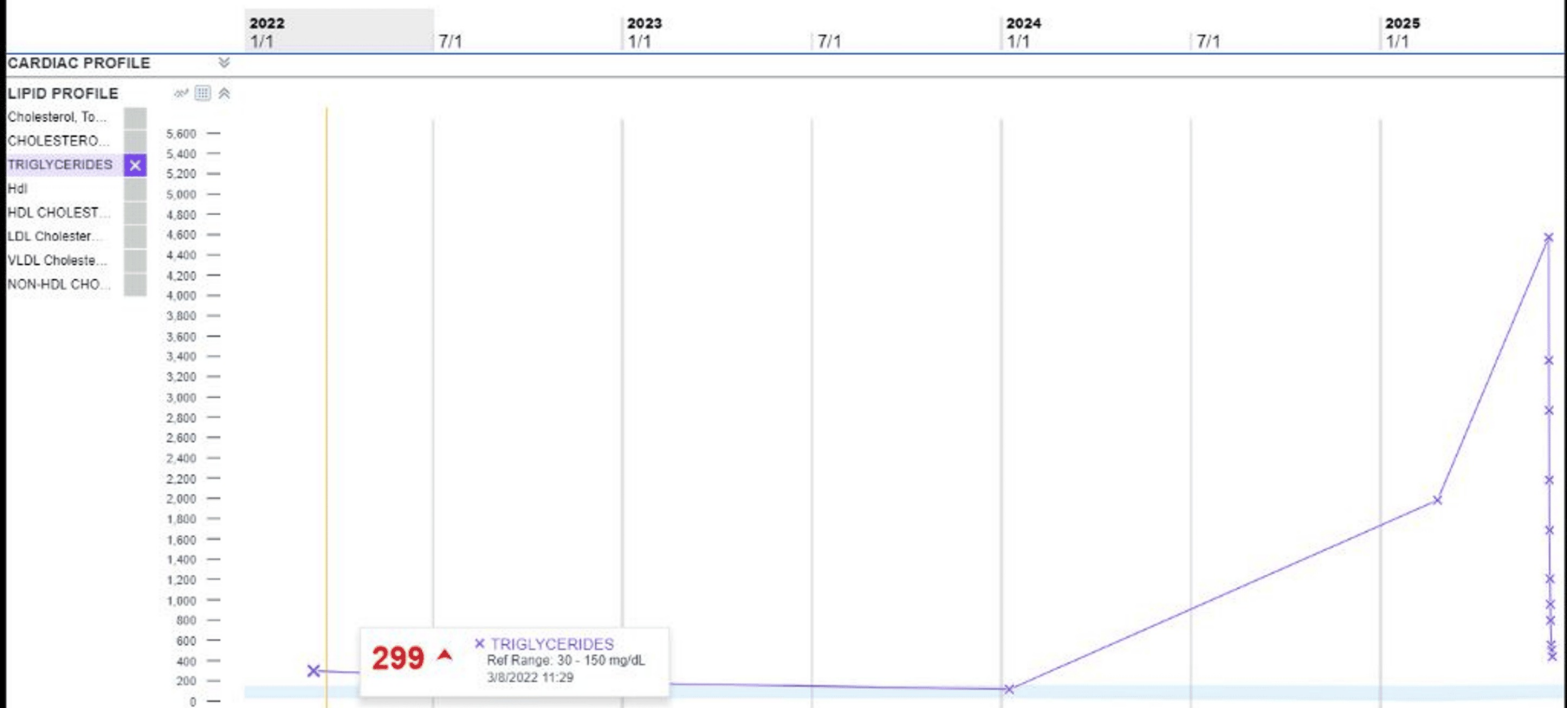
Downtrending triglyceride levels

The patient was admitted to the medical ICU for HTGP and initiated on an insulin infusion, with subsequent downtrending of triglycerides (Figure 3), and transitioned to fenofibrate. By hospital day five, insulin therapy was discontinued, and the patient was clinically stable and tolerating oral intake, allowing for transfer to the medical floor and discharge home on day six. 

## Discussion

Although hypertriglyceridemia is a recognized but infrequent cause of acute pancreatitis, it remains easily overlooked in emergency settings, especially in the absence of classic risk factors such as poorly controlled diabetes or familial dyslipidemias [2-4]. In this case, HTGP was not immediately suspected based on history alone. However, the patient's visibly lipemic serum during phlebotomy prompted targeted triglyceride testing - a key diagnostic pivot. This reinforces the value of clinical vigilance and the diagnostic impact of keen visual observation, particularly when standard workups may miss uncommon causes.

Several predisposing factors likely converged in this patient. She was obese and on antiretroviral therapy (ART), both of which are known contributors to lipid metabolism disturbances. Protease inhibitors and some integrase inhibitors have been linked to elevated triglyceride levels, even in the absence of overt dyslipidemia. Interestingly, this patient’s historical lipid panels - including her most recent annual levels (Figure 2) - had consistently shown triglyceride values in the low hundreds, indicating that her severe hypertriglyceridemia was either acute or precipitated by additional metabolic stressors. This underscores the unpredictable nature of HTGP and the importance of maintaining diagnostic suspicion even when prior lab trends appear reassuring.

Management of HTGP differs fundamentally from other causes of pancreatitis, with early triglyceride-lowering therapy being central. Insulin infusion, as used here, promotes lipoprotein lipase activity and enhances triglyceride clearance [3,4]. In more severe or refractory cases, plasmapheresis may be considered [5]. Timely recognition and intervention are critical, as HTGP has been associated with higher rates of complications, including recurrent pancreatitis, organ failure, and systemic inflammation [7].

This case illustrates how subtle clinical cues - such as the appearance of blood samples - can signal life-threatening metabolic conditions. It also highlights the need for broader awareness among emergency clinicians that triglyceride-induced pancreatitis may present without classic red flags. Routine lipid panels are seldom ordered in acute abdominal pain workups, but as this case shows, they can make all the difference when guided by thoughtful observation.

## Conclusions

This case of HTGP highlights how thoughtful clinical observation - specifically noting lipemic serum during phlebotomy - can lead to early identification of a rare but serious diagnosis. Despite the absence of classic risk factors such as diabetes or known dyslipidemia, this patient’s obesity and use of antiretroviral therapy may have contributed to the acute metabolic derangement. The decision to pursue a triglyceride level, guided by visual observation rather than protocol, was pivotal. Prompt recognition enabled targeted therapy, including insulin infusion and lipid-lowering agents, which led to rapid clinical improvement. This case emphasizes the enduring value of bedside acumen in emergency medicine, especially when managing common presentations with uncommon causes.
